# Third Annual Report of the Chicago Charitable Eye and Ear Infirmary

**Published:** 1861-07

**Authors:** 


THIRD ANNUAL REPORT OF THE CHICAGO CHARITABLE EYE AND EAR INFIRMARY. —Presented by the Board of Surgeons, for the year ending May 1, 1861. Chicago.

In presenting this Report, the Surgeons take occasion to state that they find many reasons for renewed encouragement in their labors. The number of patients applying for aid has steadily continued to increase. Benevolent individuals in this and the adjoining States have so far shown their confidence in the Institution, as to send poor patients to the Infirmary, at their own expense, for treatment. Physicians throughout the State have, in various ways, testified their interest in the success of the Infirmary; and all our intelligent citizens, to whom its beneficent objects have become known, generously tender their aid in its continued support.

The treatment has been very gratifyingly successful; and much suffering, destitution and distress has been alleviated through the instrumentality of the Infirmary.

Quite a large proportion of the patients at the Infirmary has been poor children, with whom diseases of the eye are too often, from fear of expense on the part of their parents, left without treatment, till vision has become permanently injured, and not unfrequently destroyed.

The Surgeons earnestly seek to impress upon the minds of the trustees and of the public, that the great object of Charitable Eye Infirmaries is the *prevention* of calamities—the most terrible that can befall a human being. By furnishing the poor with gratuitous treatment for diseases of the eye, they remove the fear of expense, and thus encourage such patients to apply for medical aid in the earliest stages of disease, when most easily relieved. In this way they prevent not only blindness, but idleness and pauperism, with all their attending evils.

Prevention of these evils is more politic, vastly more economical, and more in accordance with an enlightened humanity, than efforts to alleviate their sad and deplorable effects, after they once exist. Even on the ground of economy alone, is it not a matter worthy the careful consideration of all tax-paying citizens, that more than four thousand poor patients are annually treated at the New York Eye Infirmary, at an expense of only about *one dollar each*, while a class of less than one hundred pupils is maintained in the Blind Asylum of this State at an expense of not much less than *two hundred dollars each?* And, too, a very large proportion of pupils in all blind asylums become blind from inflammatory diseases of the eye, many of which, it is stated on good authority, could have been relieved by proper treatment in their early stages.

Especial attention is called to the fact, that in this State, as also in the entire Northwest, there is a very large number of poor patients afflicted with diseases of the eye, who are almost entirely without suitable medical treatment, unless furnished by an institution like this.

The Surgeons, in concluding their report, of which the above is a very full abstract, would commend the Infirmary anew to its friends and the public, and earnestly ask such aid from the benevolent and affluent, as its increasing demands and extending usefulness require.

Dr. Edward L. Holmes, attending surgeon, who has made diseases of the eye and ear a speciality, is, we are glad to know, abundantly satisfied with the success of the Infirmary, modes of treatment, etc.; and deserves high praise for his energy and perseverance in its conduct. Two hundred and eighty-eight patients were treated during the year; of which 237 were for diseases of the eye, and 51 of the ear—making, in all, 580 cases treated since the establishment of the institution.

